# Immune responses result in misdiagnosis of *Schistosoma japonicum* by immunodiagnosis kits in egg-positive patients living in a low schistosomiasis transmission area of China

**DOI:** 10.1186/1756-3305-7-95

**Published:** 2014-03-06

**Authors:** Shu-Ying Xie, Min Yuan, Min-Jun Ji, Fei Hu, Zhao-Jun Li, Yue-Min Liu, Xiao-Jun Zeng, Hong-Gen Chen, Hai-Wei Wu, Dan-Dan Lin

**Affiliations:** 1 Jiangxi Provincial Institute of Parasitic Diseases, Nanchang, Jiangxi 330046, China; 2 Department of Pathogen Biology, Nanjing Medical University, Nanjing, Jiangsu 210029, China; 3 Center for International Health Research, Rhode Island Hospital, Brown University Medical School, Providence, RI, USA; 4 Department of Pediatrics, Rhode Island Hospital, Brown University Medical School, Providence, RI, USA

**Keywords:** *Schistosoma japonicum*, Humoral immune response, Cellular immune response, Antibody response, China

## Abstract

**Background:**

In recent field surveys, we failed to detect the presence of specific antibody against *Schistosoma japonicum* in some egg-positive patients by commonly used immunodiagnostic kits. To find out whether low levels of specific antibody truly exist among egg-positive individuals and elucidate the underlying immune mechanisms, we carried out a cross-sectional epidemiologic study in a *S. japonicum* low transmission endemic area of Poyang Lake region, China and compared the humoral and cellular immune characteristics between *S. japonicum* high and low antibody responders.

**Methods:**

Kato–Katz thick smear assay was used to determine the schistosomiasis status of 3,384 participants residing in two Poyang Lake region villages, Jiangxi, China. Among the 142 stool egg-positive participants, we identified low and high *S. japonicum* antibody responders with soluble egg antigen (SEA) and adult worm antigen (AWA) specific IgG levels by adopting ROC curve analysis. To compare the humoral and cellular immune responses between high and low *S. japonicum* antibody responders, serum specific antibody levels as well as the percentage of T lymphocyte subpopulation in PMBC, and cell stimulated cytokines (IFN- gamma and interlukin-10) were detected.

**Results:**

Eight *S. japonicum* egg-positive participants were defined as low antibody responders. Although the percentage of CD3^+^T cells in low responders was slightly higher and the percentage of CD4^+^ T cells, CD8^+^ T cells, the ratio of CD4^+^/CD8^+^ and CD4^+^ CD25^+^ Treg cells were lower than those in high responders, the differences between the two groups were not significant (P > 0.05). AWA -stimulated interlukin-10 level was significantly higher in high responders, while other cytokines did not show differences between two groups. For antibody profiles, except AWA specific IgA, significant differences of each antibody isotype between low and high responders were detected (P < 0.05).

**Conclusions:**

Our study confirmed that there are *S. japonicum* antibody low responders among schistosome egg-positive residents in *S. japonicum* low-transmission areas in China. Thus, mis-diagnosis using immune-diagnosis kits do exist. Significant differences of responding antibody levels between low and high responders were detected, while no major cellular response changes were observed.

## Background

Schistosomiasis is a serious public health problem and hampers social and economic development in endemic areas. During the past 60 years, China has made remarkable achievements in controlling the endemicity of schistosomiasis japonica. Since 2006, with the full-scale implementation of integrated strategy emphasizing on infectious source control, the prevalence of schistosomiasis continuously decreased toward the fulfillment of the national mid- and long-term control program for schistosomiasis (2004-2015). In 2008, the epidemic control criteria have been met in all endemic areas in China, (i.e., schistosomiasis infection rate of both human and livestock are less than 5%) [1,2]. Nevertheless, schistosomiasis remains endemic in 8 provinces in China with relatively low prevalence and intensity. There are 28.7 million diagnosed patients (including 2.3 million of advanced cases) and 245 million of people at risk [3]. Effective control of schistosomiasis has become the urgent need for improving the health care of people living in the *S. j* areas.

Accurate diagnosis is crucial for schistosomiasis control and prevention. In China, parasitological and immunological assays are the main diagnostic approaches. It is well-known that parasitological examinations miss cases in low intensity infections [4-6]. For this reason, immunological assays have been widely used as a supplement or alternative method for diagnosis due to its high sensitivity and simplicity. Needless to say, immunogical assay has played an important role in screening target populations for chemotherapy and surveillance [7-11].

Despite the previously mentioned advantages of immunodiagnosis, there are also disadvantages of immunoassay when applied to schistosomiasis diagnosis, i.e., it can neither discriminate active infection from past infection, nor provide accurate epidemiological information of true prevalence or transmission intensity [12-17]. False-positive results are a common problem. Interestingly, we recently noticed that in some field surveys, participants with positive stool examination results were diagnosed *S. japonicum* negative by a few commercially available *S. japonicum* immunodiagnosis kits [16,18,19]. The misclassified detection results may thus result in missing treatment targets and lead to a potential risk of disease transmission. In the present study, we aim to find out the incidence of missed cases by immunodiagnosis kits among egg-positive individuals residing in schistosomiasis foci as well as explore the underlying immune mechanisms. A cross-sectional epidemiologic study was carried out in two *S. japonicum* low transmission villages in the Poyang lake region of China. Specific antibodies, T lymphocyte subsets and cytokines responses were measured and compared between high and low antibody responder groups.

## Methods

### Study area and participants

The study was conducted in two villages, namely Xinhua and Zhuxi in Xingzi county of Jiangxi province, China. The study foci are lake-type endemic areas of schistosomiasis. Both villages located on the northern shore of Poyang Lake, downstream of Gan River with similar geographical settings. Residents are of similar socioeconomic status, life style and occupation. Agriculture, aquaculture and migrant working are their main income source. About 50% of young people under the age of 40 leave their village home and make a living in the urban cities whilst only visiting home about once a year. Villagers exposed to cercariae contaminated water due to daily activities and working behavior, such as fishing, washing clothes etc. Wetland Dongwugui and Zhuqi are the 2 main water sources that are adjacent to the village land with snail breeding areas of 169.8 hm^2^ and 98.6 hm^2^ respectively. The main infectious sources are fishermen and cattle.

The ethics committee of the Jiangxi Provincial Institute of Parasitic Diseases approved this study (ID 201002003). The study objectives and procedures were explained to all residents and only those granting informed consent were enrolled in the study. Praziquantel (40 mg/kg) treatment was provided to all participants who were stool egg-positive by Kato-Katz examination during baseline survey.

### Faecal examination

All villagers over 5 years old were invited to participate in the baseline survey. Each was asked to provide three stool samples at intervals of 3–5 days. Every submitted stool sample was used to make 9 slides following the instruction of Kato–Katz thick smear technique for *S. japonicum* egg detection. The slides were examined back-to-back by three qualified microscopists. *S. japonicum* infection intensity was recorded as eggs per gram feces (namely EPG). Each egg-positive slide was confirmed by at least two of the qualified microscopists. Individuals with confirmed egg-positive stool results were further recruited as study subjects.

Forty-three healthy individuals (without history of *S. japonicum* exposure, and confirmed *S. japonicum* negative by both serologic detection and stool examination) from a non-schistosomiasis epidemic area, Ruijin city of Jiangxi province, were interviewed and recruited as negative controls.

### Blood collection

Venous blood was collected from 43 negative controls and 142 egg-positive participants before praziquantel treatment. About 2 mL blood was drawn into a serum separation tube for antibody assays, while 10 ml blood was drawn into heparinized vacutainer tubes for PBMC isolation and cell stimulation assays.

### Antigen preparation

*S. japonicum* (1500 ± 100 cercariae/rabbit) infected rabbit’s livers were used to isolate schistosome eggs. The livers were cut into smaller cubes and triturated for isolation of eggs. Purified eggs were suspended in 0.9% ice-cold saline and homogenized on ice. After repeated freeze/thaw treatment, the homogenate was ultracentrifuged at 10,000 g for 30 minutes at 4°C, clear supernatants were collected as soluble egg antigen (SEA). Adult *S. japonicum* worms (mixture of males and females) were lyophilized and triturated, and then suspended in 0.9% saline and homogenized on ice. After repeated freeze/thaw treatment, the homogenate was centrifuged at 10,000 g for 30 minutes at 4°C and then clear supernatants were collected as adult worm antigen (AWA). Protein concentration was determined by bicinchoninic acid assay (Pierce, Rockford, IL, USA). The concentration of endotoxin in these antigen preparations was below 0.03 EU/ml, assayed by a timed gel endotoxin detection kit (Sigma, St. Louis, MO) according to the instructions provided by the manufacturer.

### Diagnosis of *S. japonicum* infection by immuno-diagnostic kits

All serum samples were tested in parallel with five schistosomiasis immuno-diagnostic kits that are commercially available in China. The five kits are ELISA-based kits (Shenzhen Combined Biotech Co. Ltd., China. batch number 20120315, referred to as M1 in this paper), DIGFA kit (Shenzhen Combined Biotech Co. Ltd., China. batch number 20120315, referred to as M2), DDIA kit (Wuxi Saide co., Ltd., China, batch number 1203121, referred to as M3), IHA kit (Anhui Anji medical Co. Ltd., China, batch number 20110418, referred to as M4), and IHA kit (Jiangxi Huanpo Co. Ltd., China, batch number 20111220, referred to as M5). All kits detect levels of *S. japonicum* soluble egg antigen (SEA) specific antibody by different means. Tests were performed following manufacturers instructions in our laboratory. Blank, positive and negative control sera were set up simultaneously with samples at each test. Results of the M1 kit were quantified at OD_450_ by E-max mircoplate reader (Molecular Devices, USA), while results of kits M2, M3, M4, and M5 were determined by visual inspection.

### *S. japonicum* specific antibody measurement

*S. japonicum* adult worm antigen (AWA) and soluble egg antigen (SEA) specific IgG, IgG1, IgG2, IgG3, IgG4, IgA, IgE and IgM antibody isotypes were detected with indirect enzyme-linked immunosorbent assay (ELISA). Briefly, 96-well ELISA plates (Costa, USA) were coated with AWA (3 ug/well) or SEA (4 ug/well) in carbonate buffer (pH 9.6), and stored overnight at 4°C. Plates were washed once with phosphate-buffered saline containing 0.05% Tween-20 (PBS-T, pH 7.2), and blocked with 5% (w/v) non-fat milk in PBS at 37°C for 1 h. Serum samples were diluted in 1:100 with PBS for detection of IgA, IgG2, IgG3, and IgG4, 1:200 for IgG1 and IgG, 1: 10 for IgE, and 1:400 for IgM. After addition of 100 ul diluted serum to each well, plates were incubated at 37°C for 2 h. The plates were then washed 3 times with PBS-T, followed by incubation at 37°C for 1 h with 100 ul of the HRP-labeled goat anti-human IgG, IgG1, IgG2, IgG3, IgG4, IgA, IgE and IgM antibody (SouthernBiotech, USA) at appropriate dilution in PBS. Chromogenic substrate TMB (3, 3′, 5, 5′-tetramethylbenzidine) was added after incubation and 4 times wash with PBS-T. OD_450_ was determined by E-max microplate reader. All serum samples were assayed in duplicate, and the normalization was performed to suppress plate-to-plate variation by using internal standards [20]. For each test, blank, positive and negative control sera were run simultaneously with testing samples.

### Defining low *S. japonicum* antibody responders

The receiver operating characteristic (ROC) curve was used to determine the optimal threshold of schistosome antibody (SEA-, AWA-IgG) detected by ELISA. Samples are composed of the schistosome infected individuals and non-infected healthy persons. After ROC curves were constructed, the cut-point that achieved the maximum Youden index (sensitivity + specificity-1) was referred to as optimal threshold. Those with both SEA and AWA-specific IgG levels lower than their optimal thresholds were defined as low *S. japonicum* antibody responders. Likewise, those with both antibody levels higher than the optimal thresholds are defined as high *S. japonicum* antibody responders [21].

### Peripheral blood mononuclear cell (PBMC) stimulation and cytokine measurement

PBMCs were prepared from each of the subjects using Ficoll-Hypaque separation (TBDscience, China) according to the instructions. Briefly, 10 ml whole blood were centrifuged at 400 g for 30 min at 15°C in the density gradient. The cells were re-suspended in RPMI 1640 medium (Thermo, USA) with 10% fetal bovine serum (Thermo, USA). For cytokine detection, a total of 2 × 10^6^ cells in 1 ml of medium were plated in triplicate into a 24-well plate (Costa, USA) and incubated at 37°C with 5% CO_2_ for 72 h with different stimulation schemes. The stimuli are AWA (30 ug/ml), SEA (10 ug/ml) and Phytohemagglutinin (PHA, 10 ug/ml) (Sigma, USA). RPMI with cells only was placed as blank control. Approximately 800 ul of supernatants were collected from each set of stimulation and stored at -70°C for cytokine measurement.

A monoclonal antibody-based capture ELISA kit from BioLegend (San Diego, USA) was used to detect IFN- γ and IL-10 in culture supernatants according to manufacturer’s instructions. The standard curve was made by adding serial dilutions of the standards at 1:2 fold to the 96-well plate in duplicate. The concentration of standard curve ranged from 7.8-500 pg/ml for IFN-γ and 3.91-250 pg/ml for IL-10. The standard curve was used for IFN-γ and IL-10 level quantification [22].

### T lymphocyte subpopulation detection

For cell surface staining and analysis, 1 × 10^6^ PBMCs were incubated with the specific antibodies (eBiosciences, San Diego, USA) for 30 min at room temperature. The antibodies used included anti-CD3-APC for T cells, anti-CD3-APC and anti-CD4-PE or anti-CD8-FITC for CD4^+^ or CD8^+^ T cells. Cells were sorted by a FACSCalibur flow cytometer (Becton Dickinson, San Jose, USA) and the data was analyzed using CellQuest software (Version 1.22, Becton Dickinson).

For detecting the proportion of CD4^+^CD25^+^ Treg cells, intracellular FoxP3 staining was performed using Human Regulatory T Cell Staining Kit (eBioscience, San Diego, USA) according to the manufacturer’s protocol. Briefly, 1 × 10^6^ PBMCs were labeled with anti-CD4-FITC and anti-CD25-APC, followed by fixation and permeabilization with Cytofix/Cytoperm medium (BD Pharmingen, San Diego, USA). Then PBMC were intracellularly stained with Phycoerythrin (PE)-conjugated anti-Foxp3 or IgG2a isotype control antibodies. The CD25^+^Foxp3^+^ Treg cells were analyzed after gating on the CD4^+^ T cells.

### Statistical analysis

#### *Data processing*

For *S. japonicum* infection status diagnosed by immuno-diagnostic kits M1 to M5, results were interpreted as positive and negative according to the manufacturer’s instructions.

For *S. japonicum* specific antibody measurement, we normalized antibody levels detected on different plates using I-STOD method. In short, one aliquot of a mixture of serum with high *S. japonicum* antibody titers were used on every ELISA plate with serial dilutions serving as standard curves. The OD_450_ value of each sample was converted into relative concentration against that of serum standards according to I-STOD calculation prior to data analysis [23].

#### *Data analysis*

All data are reported as the mean ± SD. Data analysis was performed using SPSS (Version 11.5, IBM, USA). After low *S. japonicum* antibody responders were identified, controls were matched by age, sex, infection intensity, liver B-ultrasound results and history of *S. japonicum* treatment at 1:1 ratio. All data were evaluated by one-way analysis of variance. Student’s *t*- test was used for pairwise comparison. Statistical significance was considered as *P* < 0.05.

## Results

### Characteristics of *S. japonicum* egg-positive patients

A total of 3,384 individuals from the 2 study villages consented and completed their 3-stool sample submission for Kato-Katz examination. Among them, 142 blood samples were collected from 182 egg-positive participants. The age of the study population ranged between 5-70 years, with an average of 39 years in males and 44 years old in females participants. Among them, 36.23% are either fishermen or boatmen. The arithmetic mean of EPG is 33.44, while the highest EPG is 960. Infection intensities are low (EPG < =20) in more than 80% of egg-positive patients. Details shown in Table 1.

**Table 1:** **Basic information of *S. japonicum *egg-positive participants**

**Age groups**	**No. by gender**		**No. by occupation**				**No. by infection intensity**		
	**Male**	**Female**	**Peasants**	**Fishermen and boatmen**	**Students**	**Others**	**EPG < =20**	**20 < EPG < =100**	**EPG > 100**
5-	24	5	0	0	28	1	21	4	4
15-	7	1	0	0	5	3	4	3	1
20-	3	2	0	1	0	4	5	0	0
30-	8	9	1	12	0	4	14	1	2
40-	16	21	4	25	0	7	32	3	2
50-	14	7	8	10	0	3	16	4	1
60-	18	7	18	4	0	4	22	1	2
Total	90	52	31	52	33	26	114	16	12

### Performance of schistosomiasis immuno-diagnostic kits in egg-positive cases

Serum samples from the *S. japonicum* egg-positive individuals were detected by the five commercially available schistosomiasis japonica immuno-diagnostic kits. Table 2 shows that the mis-diagnosis rate (false negative, i.e. egg positive but sero-negative) of M1, M2, M3, M4, and M5 kits are 5.00%, 9.49%, 11.43%, 14.63% and 15.94% respectively. That is, 7, 13, 16, 18 and 22 out of 142 egg-positive cases are misdiagnosed by each of these kits respectively. Most (94.4%) of the missed patients are at low infection intensity (EPG < =20).

**Table 2:** Performance of Schistosomiasis immuno-diagnostic kits in egg-positive participants stratified by infection intensity

**Diagnostic kits**	**EPG < =20**			**20 < EPG < =100**			**EPG > 100**			**Total**		
	**No. of detection**	**No. of missed cases**	**Missing rate (%)**	**No. of detection**	**No. of missed cases**	**Missing rate (%)**	**No. of detection**	**No. of missed cases**	**Missing rate (%)**	**No. of detection**	**No. of missed cases**	**Missing rate (%)**
M1	112	7	6.25	16	0	0.00	12	0	0.00	140	7	5.00
M2	109	13	11.93	16	0	0.00	12	0	0.00	137	13	9.49
M3	112	16	14.29	16	0	0.00	12	0	0.00	140	16	11.43
M4	101	17	16.83	12	0	0.00	10	1	10.00	123	18	14.63
M5	110	22	20.00	16	0	0.00	12	0	0.00	138	22	15.94

### Identification of *S. japonicum* low and high responders

**Figure 1:**
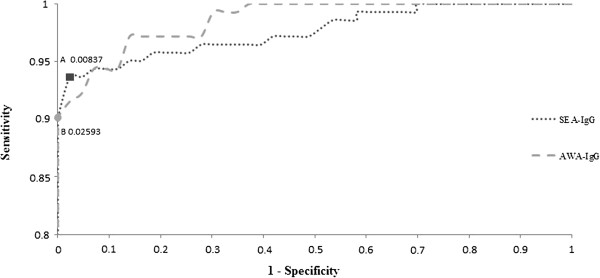
**The receiver operating characteristic (ROC) curves of SEA-IgG and AWA-IgG.** The ROC curve analysis was performed in order to determine the cut off values of SEA- and AWA-IgG. Youden indexes were maximized at point A (0.00837) and B (0.02593) respectively on ROC curves.

Of the 185 serum specimens that were tested for SEA- and AWA- specific antibodies, 142 were from schistosome egg-positive paticipants and 43 from healthy individuals living in non-endemic areas of schistosomiasis. ROC curves were generated according to the relative antibody concentration of SEA- and AWA-specific IgG. Stool examination results were used as gold standard to calculate the specificity and sensitivity of ELISA method. It shows that when sensitivity of the SEA-IgG antibody was 93.7% and specificity was 97.7%, the largest Youden index reached 0.91, thus the corresponding point of contact was set as the optimal threshold value of 0.00837 for SEA-IgG positive response (responders) on the ROC curve (Figure 1, point A). For AWA- IgG, largest Youden index of 0.90 appears when sensitivity was 90.1% and specificity was 100%. Thus, the optimal threshold value for AWA-IgG responders was set as 0.02593 (Figure 1, point B).

When both the relative concentration of SEA-IgG and AWA-IgG were less than the optimal threshold values (0.00837 and 0.02593), the egg-positive participants were defined as low *S. japonicum* antibody responders*.* Vice versa, high responders are those with both antibody levels higher than the optimal threshold values. Accordingly, eight low responders were identified from the 142 egg-positive patients. These low responders were aged from 6 to 79 with the average age of 42 years old. Occupations of these low responders varied from students, fishermen, housemakers to businessmen. Seven out of the eight low responders had histories of contaminant water exposure and praziquantel treatment. The highest EPG among these low responders was 13, while the majority subjects (7 out of 8) have very low EPG of 1-3. All low responders had various levels of liver parenchyma damage. Among them, one had hepatomegaly and 4 had splenomegaly as examined by B-ultrasound. However, when diagnosed by the five commercially available kits, two out of these low responders were defined as schistosomiasis negative by all kits, and the other six were considered as negative by at least one kit (Table 3).

**Table 3:** **Characteristics of *S. japonicum *antibody low responders**

**Study ID**	**Gender**	**Age**	**Occupations**	**EPG**	Kits that diagnosed negative*	B-ultrasound examination**			**History of schistosomiasis treatment (times)**
						**Liver fibrosis**	**Hepatomegaly**	**Splenomegaly**	
1702106	F	14	Student	2	1,2,3,4,5	+	-	-	1
1505502	F	70	Housework	2	1,2,3,4,5	+	-	+	3
1305401	F	47	Businessman	2	2,3,4	+	-	-	2
1801206	F	27	Businessman	3	1,2,3,4,5	+	+	+	2
1301106	F	44	Fisherman	2	2	+	-	+	8
1105001	M	47	Businessman	13	1,2,3,4,5	+	-	-	1
1506601	M	79	Housework	1	2,3,4,5	+	-	+	20
4502805	M	6	Student	3	1,2,3,4,5	+	-	-	0

*The number of the kits that diagnosed the subject negative is listed. For examples, 1, 2, 3, 4, 5 means that *S. japonicum* immuno-diagnostic kits of M1、M2、M3、M4、M5 all tested the subject as *S. japonicum* negative.

**B-ultrasound examination results are recorded as “+” for positive and “-” for negative.

### Comparison of immunological characteristics between low and high responders

Immunological characteristic comparisons were carried out between low and high responders. The eight subjects in the high responder group were identified from the pool of high responders with matched age, gender, *S. japonicum* infection intensity, liver B-ultrasound results and *S. japonicum* treatment history to the eight low responders at 1:1 ratio.

#### Comparison of *S. japonicum* specific antibody isotype level between low and high responders

**Figure 2:**
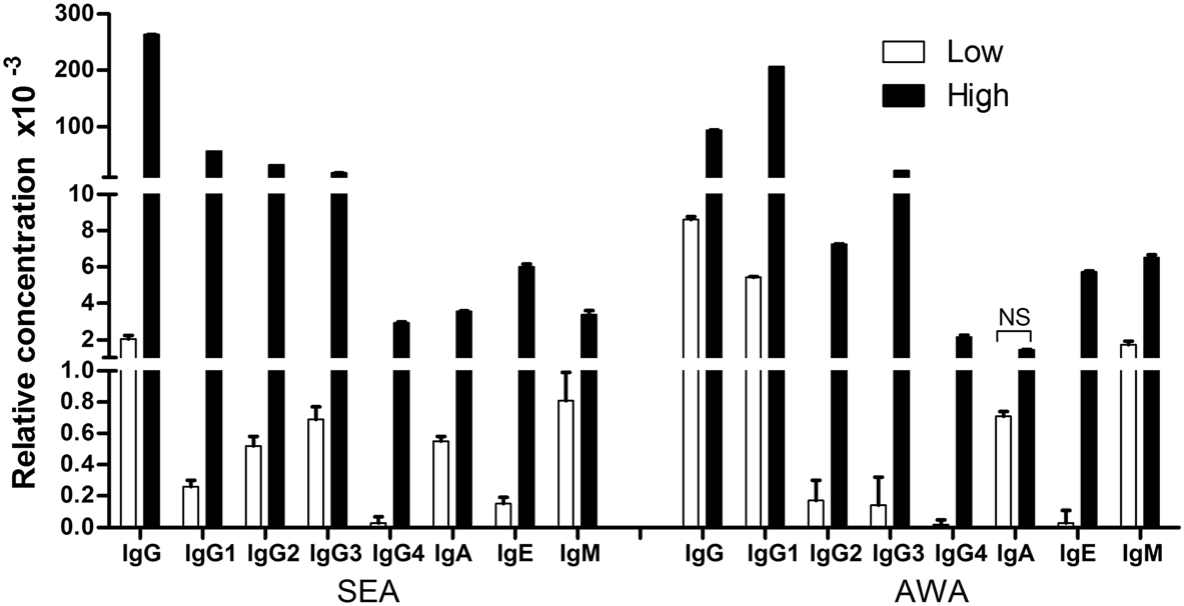
**Schistosome-specific antibody isotype levels in low and high antibody responders.** Serum levels of schistosome-specific IgG, IgG1, IgG2, IgG3, IgG4, IgE, IgM, IgA were analysed by ELISA. Relative concentrations of antibody isotypes were compared between the high responders and low responders. NS, no significance. Open square bars represent mean of relative antibody concentration of low responders, black square bars represent mean of relative antibody concentration of high responders. Error bars represent SEMs.

Figure 2 shows significant differences (*P* <0.05) between low and high responder groups for each antibody isotype except AWA-IgA.

#### Comparison of T cell subgroup percentage and S. japonicum antigen stimulated cytokine levels in peripheral blood between high and low responders

**Figure 3:**
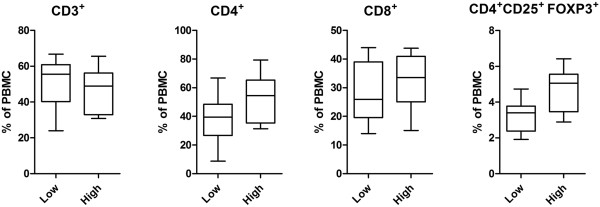
**Proportions (%) of T lymphocytes in PBMCs of low and high antibody responders.** Proportions of CD3^+^, CD4^+^, CD8^+^, CD4^+^ CD25^+^FoxP3^+^ T cells (expressed as % of PBMC) were compared between low and high responder groups. Differences were analysed by multivariate analysis after accounting for the effect of sex and age. Data are presented as whiskers boxplot (10-90 percentiles). No significant difference was detected between the two groups.

Compared to the high responder group, the percentage of CD3^+^ T cells in the low antibody responder group was slightly higher (51.54 ± 14.95 vs. 45.49 ± 13.33), but with no significant difference (*P >* 0.05). The percentage of CD4^+^ T cells, CD8^+^ T cells, the ratio of CD4^+^/CD8^+^ and CD4^+^ CD25^+^ Treg cells were lower in low responders. The difference between two groups were not significant *(p >* 0.05) (Figure 3).

**Figure 4:**
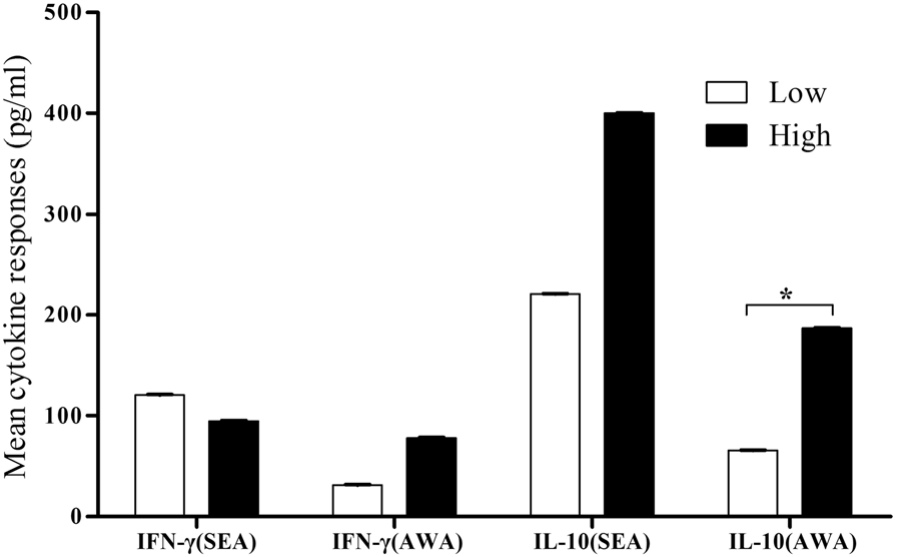
**Comparison of SEA- and AWA-stimulated IFN-γ and IL-10 between low and high antibody responders.** Open square bars represent mean of relative antibody concentration of low responders, black square bars represent mean of relative antibody concentration of high responders. Error bars represent SEMs. *, significant difference (*P* <0.05).

No significant differences of PBMC- secreted IFN-γ or IL-10 levels were observed between low and high responders *(p >* 0.05), except for AWA stimulated IL-10 (*P* <0.05, See Figure 4).

## Discussion

The human immune system functions through cellular and humoral immune coordination. Antibody production is the result of humoral responses to antigen induction [24]. For schistosomiasis diagnosis, the traditional strategies rely on the detection of parasite eggs in stool samples. Theoretically, schistosomiasis patients diagnosed by egg examination using microscopy should be positive in antibody detection assays, considering that the sensitivity of antibody detection assays have been shown to be higher than by stool examination [25]. However, studies in recent years hinted that a certain number (5.5%-30.14%) of human schistosomiasis cases (egg positive in stool examination) might have been missed by antibody detection diagnosis especially when prevalence and infection intensity were at low levels [18-20,26]. In the current study, up to 17 out of 142 confirmed schistosomiasis cases found in the 2 schistosomiasis low-endemic villages were mis-diagnosed when examined by five commercial schistosomiasis immuno-diagnostic kits in parallel, and the rate of false negatives for each kit is 5.00%-15.94%.

Reliable detection methods, suitable choice of detection targets and correct result interpretation are important factors for achieving an accurate diagnosis of schistosomiasis. Currently, antibody detection kits for schistosomiasis diagnosis developed and commonly used in China can be classified into 3 categories according to the type of immune assays, such as IHA, DIGFA, and ELISA. Among them, ELISA is the most widely used assay for its high sensitivity and stability [8]. IgG antibody is a widely used detection target in immune tests because it is one of the important antibody isotypes in human humoral responses. ROC curve method had been widely accepted for its scientific nature, objectivity and accuracy in determination of threshold value [21,27]. For these reasons, we use the level of total schistosome antigen specific IgG antibody to evaluate the humoral response of schistosomiasis patients, and threshold value of ELISA assays were determined using ROC curve. Therefore, participants with serum concentration of SEA or AWA specific IgG lower than the threshold value would be considered as low antibody responders.

A total of eight egg-positive patients were determined as low antibody responders with *S. japonicum.* Their age distribution suggested that elder peoples with partial immunity were prone to have a low *S. japocium* antibody response. In addition, B-ultrasound revealed hepatic fibrosis in all low responders. Hepatic fibrosis is considered to be induced by the immune response to schistosome eggs [28] and regulated by cytokines and chemokines [29,30]. Our findings in the present study indicate that the immunoregulatory effects might play a role in the immune response profile among these patients.

Immune non-responders or weak responders have previously been identified in Hepatitis B vaccinated populations, lymphatic filariasis patients or untreated tuberculosis patients [31-35]. The mechanisms involved have not been fully elucidated so far. Previous studies demonstrated that immune function and genetic factors had played an important role in people with low immune responses. Among all the factors affecting the immune response to Hepatitis B vaccination, imbalance of cellular immune function has proved to be the most important one. Several reports have described schistosome infection correlated with immune responses regulation [36,37]. Immune response modulation after schistosome infection was associated with an increased number of regulatory T cells, and high levels of regulatory cytokines, such as IL-10 [38,39]. To investigate the immune characteristics of those *S. japonicum* antibody low responders, we compared humoral and cellular responses between the low responders and a matched high antibody responder group. The results showed SEA and AWA specific antibody isotypes in low responder groups were significantly lower, which indicates that differences do exist extensively in humoral immune response between the two groups. However, the data also showed that no significant differences were found in T cell populations including CD4^+^CD25^+^T regulatory cells. Tregs are recruited by schistosome eggs and involved in down-regulation of host immune responses [38]. We also compared the level of *S. japonicum* antigen stimulated IFN-γ and IL-10. IFN-γ is a Th1 cytokine and it can induce phagocytosis of macrophages at an early stage of infection. IL-10 is an important Th2 cytokine, which also plays a critical immunoregulatory role in the protection against hepatic fibrosis in human schistosomiasis [29]. Our study revealed no change of IFN-γ levels between low and high responder groups, but decreased levels of AWA stimulated IL-10 presented in low responder group. The diminished release of IL-10 was also reported in hepatitis B vaccination nonresponders [40].

Although decreased specific antibody levels were detected in low *S. japonicum* antibody responders, the sample size was too small to confirm the immune suppression in these patients. It is noteworthy that *S. japonicum* infection intensity in the 8 low antibody responders was quite low, EPG are only 1-3 in 7 cases, 1 has the EPG of 13. Keeping in mind that this was based on 3 stools of totally 27 slide readings, which is more extensive than regular stool parasitology examinations. Thus, we speculate that low antibody response or mis-diagnosis of schistosomiasis by some diagnostic kits may be the outcome of low antibody titre in the peripheral blood circulation after antigen-antibody complex has formed in blood of patients with low parasite burden [41]. To precisely delineate the immune mechanism in low *S .japocium* antibody responders, further investigation is warranted in an expanding study population.

## Conclusions

Our study confirmed that there are *S. japonicum* antibody low responders among schistosome egg-positive residents in *S. japonicum* low-transmission areas in China. Thus, mis-diagnosis using immune-diagnosis kits does exist. Significant difference of antibody responding isotypes between low and high responders were detected, while no major changes on cellular responses were observed.

It is important to learn from this study that antibody detection assays are limited in the schistosomiasis field with low infection intensity. New approaches for *S. japonicum* detection are necessary for avoiding missing cases caused by low antibody responses.

## Abbreviations

EPG: Eggs per gram; AWA: Adult worm antigen; SEA: Soluble egg antigens; ELISA: Enzyme-linked immunosorbent assay; SPSS: Statistical product and service solutions; PBMC: Peripheral blood mononuclear cell; AIDS: Acquired immune deficiency syndrome; DIGFA: Dot immunogold filtration assay; DDIA: Dipstick dye immunoassay; IHA: Indirect hemagglutination assay; PBS: Phosphate buffer saline; HRP: Horse radish peroxidase; TMB: 3,3′,5,5′- tetramethylbenzidine; ROC: Receiver operating characteristic; PHA: Phytohaemagglutinin; FITC: Fluorescein isothiocyanate; APC: Allophycocyanin; PE: P-phycoerythrin; I-STOD: Improved standardization method for optical density; OD: Optical density; SD: Standard deviation.

## Competing interests

The authors declare that they have no competing interests.

## Authors’ contributions

Design of the study: DDL, HGC. Performed the experiments and data collection: SYX, MY, MJJ, ZJL, YML, XJZ. Analyzed the data: FH, MY, HWW. Drafting and revising of the manuscript: SYX, MY, ZJL, DDL, HWW. All authors read and approved the final version of the manuscript.
